# 

**Published:** 1917-06-02

**Authors:** 


RATES OF SUBSCRIPTION: Twelve months, 6/6; Six months, 4s.; three months, 2/-, post free to any address in the United Kingdom. Abroad, Twelve months, 8/8; Six months, 5/-; Three months, 2/6, post free. **THE HOSPITAL "Index" to Volume LXI., October 1916 to March 1917, is now ready, price 2½d. per copy, post free.** Address The Manager, THE HOSPITAL, "The Hospital" Building, 28/29 Southampton Street, Strand, London, W.C. 2.

**28/29 Southampton Street, Strand, London, W.C. 2.**

